# A Review of the Efficacy of the Low Fermentable Oligosaccharides, Disaccharides, Monosaccharides, and Polyols (FODMAP) Diet in Managing Gastrointestinal Symptoms Related to Cancer Treatment

**DOI:** 10.7759/cureus.56579

**Published:** 2024-03-20

**Authors:** Arwa S Almasaudi

**Affiliations:** 1 Clinical Nutrition Department, Faculty of Applied Medical Sciences, King Abdulaziz University, Jeddah, SAU

**Keywords:** gastrointestinal symptom, diet, oncology, low fodmap diet, cancer therapy

## Abstract

The low fermentable oligosaccharides, disaccharides, monosaccharides, and polyols (FODMAP) diet, designed to alleviate symptoms in individuals with irritable bowel syndrome (IBS), focuses on limiting the consumption of poorly absorbed fermentable carbohydrates known as FODMAP. These FODMAP are believed to be the primary triggers for food-related gastrointestinal symptoms in functional gastrointestinal disorders. However, there is currently insufficient direct evidence investigating the role of low FODMAP diets in cancer patients undergoing treatment. This review aims to summarize the current evidence on the low FODMAP diet and its potential implications for cancer patients in terms of treatment outcomes, alleviating gastrointestinal symptoms, and overall health. A systematic literature search was conducted using databases, including PubMed, Scopus, Google Scholar, Web of Science, and Cochrane. Five studies met the criteria for inclusion in the review, and these studies covered rectal toxicity during radiotherapy, gastrointestinal symptoms in colorectal cancer patients, acute gastrointestinal toxicity during pelvic external beam radiotherapy, symptoms in patients with radiation-induced enteropathy, and chronic gastrointestinal sequelae resulting from pelvic organ cancer treatment. The available evidence suggests that a low FODMAP diet may offer advantages in reducing rectal gas and volume during radiotherapy, alleviating diarrhea symptoms, reducing symptom deterioration, and improving quality of life. However, these studies highlight the need for large-scale randomized trials, long-term follow-up, and guidelines to establish the efficacy, safety, and implementation strategies of the low FODMAP diet in different cancer contexts and patient populations. While preliminary findings reported some possible benefits of a low FODMAP diet for certain cancer patients, rigorous studies with large sample sizes are needed to provide more robust evidence. Further research is warranted to optimize the utilization of this diet as an adjunctive intervention for managing gastrointestinal symptoms in this population.

## Introduction and background

Cancer is a significant global health issue and ranks the second leading cause of death [1]. Prostate and breast cancer are commonly found in men and women, respectively, while blood, brain, and lymph node cancer are prevalent among children [2,3]. The development of cancer is attributed to a series of genetic mutations that disrupt normal cellular functions. Carcinogenic environmental substances directly or indirectly impact the cytoplasm and nucleus of cells, leading to genetic disorders and gene mutations [4]. Other factors, such as viruses, bacteria, and radiation rays, also contribute to cancer development, accounting for approximately 7% of all cases [5]. Ultimately, cancer leads to abnormalities in the cell cycle, cellular relationships, and uncontrolled cell proliferation [6]. The manifestation of cancer signs and symptoms varies depending on the type and stage of the malignancy. Typically, patients may present with a range of clinical features, such as unexplained weight loss, fatigue, pain, alterations in the skin's appearance, changes in bowel or bladder function, dysphagia, persistent coughing or hoarseness, palpable masses or swollen lymph nodes, and inexplicable bleeding or ecchymoses [7].

Diet plays a crucial role in cancer prevention and management. Observational cohort studies suggest that up to one-third of common cancers can be prevented through dietary [8]. These studies demonstrate that certain dietary components have an influence on tumor growth, while diet can also influence metabolism and systemic inflammation that promote tumor cell proliferation and survival [9,10]. Additionally, poor dietary habits leading to obesity create an environment that promotes tumor initiation and progression [10].

Furthermore, the relationship between different types of diets, gut microbiota profiles, and cancer has been explored. A high-fiber, plant-based diet such as the Mediterranean diet may promote a healthy gut microbiota composition and reduce the risk of colorectal cancer [11]. Moreover, low-carbohydrate and ketogenic diets (KD) may have a potential role in cancer treatment, particularly for brain tumors, due to their ability to limit glucose availability to cancer cells [12] According to Weber et al.'s study, the KD has shown some promising results in cancer. The KD, characterized by high fat, low carbohydrate, and moderate protein intake, may impact the Warburg effect, a biochemical phenomenon where cancer cells predominantly rely on glycolysis for adenosine triphosphate (ATP) production [12]. Additionally, the KD has shown antitumor effects by reducing energy supplies to cells and inhibiting tumor growth [13]. Various dietary strategies have been proposed to improve cancer therapy outcomes. For example, caloric restriction has been shown to reduce cancer cell proliferation and enhance the effectiveness of chemotherapy and radiotherapy [14]. Nencioni et al. suggested that fasting or fasting-mimicking diets (FMD) can diminish the adaptability and survival capacity of cancer cells [15]. In addition, FMD enhanced the effects of chemotherapy in various cancer types. These diets have been found to heighten the sensitivity of cancer cells to chemotherapy and promote T-cell-mediated tumor cytotoxicity [16]. Nevertheless, it is crucial to consider personalized dietary plans that take into account the patient's overall health and treatment protocol [17]. While these studies show some promising results, more extensive clinical trials are needed to confirm the benefits of dietary interventions such as caloric restriction and fasting in cancer treatment.

Recently, the low fermentable oligosaccharides, disaccharides, monosaccharides, and polyols (FODMAP) diet, which focuses on limiting the consumption of poorly absorbed fermentable carbohydrates, has demonstrated promising results in managing various health conditions, particularly in patients with gastrointestinal disorders, such as irritable bowel syndrome (IBS).

The low FODMAP diet

The low FODMAP diet was first developed by researchers at Monash University in Melbourne, Australia, in early 2000, as a dietary intervention designed to alleviate symptoms of patients' functional bowel disorders, such as IBS [18]. FODMAP is an acronym for fermentable oligosaccharides, disaccharides, monosaccharides, and polyols, which are short-chain carbohydrates found in a wide variety of foods [19]. These carbohydrates are poorly absorbed in the small intestine, and when they reach the large intestine, they are fermented by gut bacteria, producing gas, and drawing water into the gut. This can lead to symptoms such as bloating, abdominal pain, diarrhea, gas, and altered bowel habits in susceptible individuals such as patients with IBS [18].

Low FODMAP diets involve a temporary restriction of high FODMAP foods, followed by a phase of structured reintroduction of those foods to determine an individual's tolerance level for these foods. Whelan et al. provided practical guidance on assessing patients with IBS and implementing and monitoring the low FODMAP diet, which consists of three stages: FODMAP restriction, reintroduction, and personalization [20].

The goal of the low FODMAP diet is to reduce gastrointestinal symptoms while maintaining a nutritionally balanced and varied diet. For example, a diet low in FODMAP restricts fermentable carbohydrates and includes various food groups, such as fresh produce, grains, cereals, legumes, nuts, seeds, dairy, meat, seafood, eggs, dairy-free alternatives, oils, fats, beverages, and snacks [21]. Fruits such as ackee, blueberries, bananas, grapes, lemons, limes, mandarins, oranges, kiwis, pineapples, passion fruits, and rhubarb are all part of the low FODMAP diet. It also includes various veggies, such as bell peppers, bok choy, green beans, parsnips, silverbeets, cucumbers, celery, eggplants, potatoes, yams, tomatoes, and zucchini. The diet allows eating quinoa, almond or rice-based milk, yogurt, ice cream, hard cheese, feta, and cottage cheese, as well as specific grains, including wheat- and gluten-free bread or cereal goods. Maple and golden syrup, as well as other sweeteners, are acceptable [22].

The diet low in FODMAP has been evaluated in a wide range of clinical studies and has been shown to be a promising treatment for symptoms associated with gastrointestinal disorders. For example, a systematic review by Zhan et al. showed a significant improvement in abdominal pain, diarrhea, abdominal bloating nausea, and fatigue after the use of the FODMAP diet among patients with IBD [23]. In addition, Molina-Infante et al. highlighted the effectiveness of the low FODMAP diet in alleviating IBS symptoms such as abdominal pain, bloating, and diarrhea, as well as its superiority over a gluten-free diet for patients with non-celiac gluten sensitivity [24]. Moreover, a study conducted by Bodini et al. in 2019 demonstrated that implementing a six-week low FODMAP diet for 60 patients with inflammatory bowel disease (IBD) resulted in a significant decrease in Harvey-Bradshaw index scores. This reduction indicates a decrease in the severity of the condition [25].

While the low FODMAP diet has shown potential benefits in managing gastrointestinal conditions such as IBS and IBD, its effects on patients with cancer have not been thoroughly investigated. Cancer patients often encounter nutritional challenges due to symptoms associated with the disease itself or the side effects of treatments such as chemotherapy and radiation. These symptoms may include nausea, vomiting, appetite loss, altered taste and smell perception, and gastrointestinal disturbances such as diarrhea or constipation [7].

However, the existing evidence is currently insufficient to validate the potential benefits of a low FODMAP diet for cancer patients. Therefore, there is a critical need for comprehensive research specifically focused on investigating whether adopting a low FODMAP diet could alleviate gastrointestinal symptoms and enhance the quality of life for cancer patients undergoing various treatment regimens. The aim of this review is to systematically gather and synthesize existing research findings on the effectiveness and safety of the low FODMAP diet in managing gastrointestinal symptoms, specifically within the context of cancer patient care. This includes examining the extent to which reducing FODMAP intake can ameliorate symptoms such as bloating, gas, and abdominal pain, which are common in cancer patients and can significantly impact their quality of life and response to cancer treatments. In addition, this review seeks to identify gaps in the current body of knowledge that need to be addressed by future research.

## Review

Methodology

Literature Search Strategy

A systematic literature search was conducted using databases, including PubMed, Scopus, Google Scholar, Web of Science, and Cochrane, to identify potentially relevant studies without date restriction. The search encompassed articles published until December 21, 2023. 

The search strategy used the following keywords ‘FODMAP’ OR ‘FODMAP’ OR ‘Fermentable, poorly absorbed, short-chain carbohydrates’, OR ‘Fermentable oligosaccharides, disaccharides, monosaccharides and polyols’, AND ‘Cancer’ OR ‘neoplasm’ or equivalent terms. Furthermore, a manual screening of the retrieved references was conducted to identify any relevant potential literature. There were no limitations regarding publication date or publication status. The protocol for this review followed the Preferred Reporting Items for Systematic Reviews and Meta-Analyses (PRISMA) guidelines.

Inclusion and Exclusion Criteria

Studies were included if they met the following criteria: involved cancer patients of any age and any cancer type and investigated the effects of a low FODMAP diet on gastrointestinal (GI) symptoms and quality of life. Studies were excluded if they were not conducted on cancer patients, did not focus on the effects of a low FODMAP diet, and did not measure symptom outcomes in cancer patients. Additionally, studies were excluded if they were not available in the English language.

Data Extraction and Analysis

The selected studies were critically appraised to assess their quality and relevance to the topic. A standardized data extraction form was created to collect relevant information from the selected studies. The following data were extracted, summarized, and presented in a descriptive manner: study characteristics, study design, participant details, intervention specifics (low FODMAP diet details), outcome measures, and key findings related to GI symptoms and quality of life.

Reporting

The PRISMA guidelines were followed. Critical appraisal was conducted using the Critical Appraisal Skills Programme (CASP) tools.

Results

**Figure 1:**
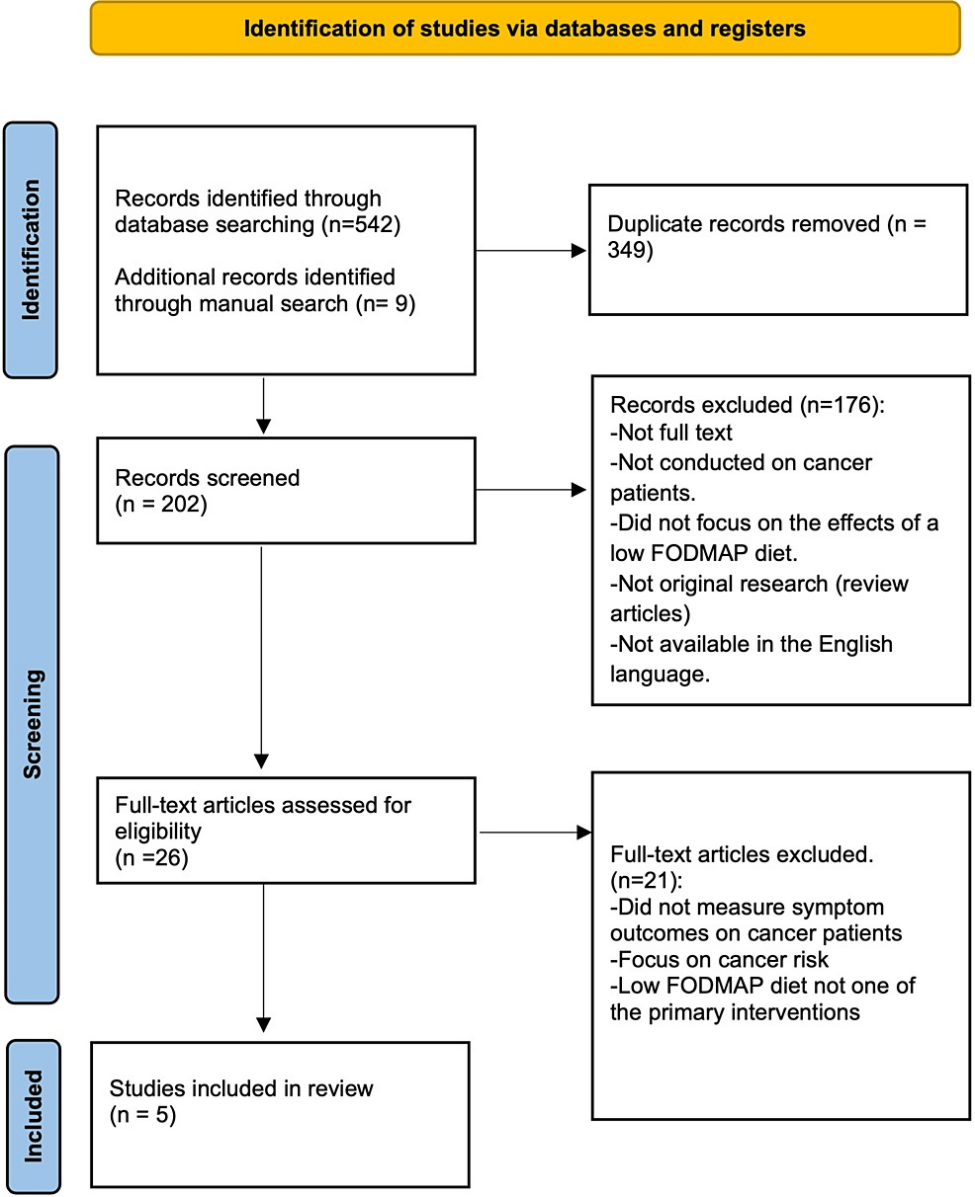
The process of obtaining the relevant papers for this study is shown in the Preferred Reporting Items for Systematic Reviews and Meta-Analysis (PRISMA) diagram.

Figure 1 illustrates the detailed process of literature selection. Initially, 542 articles were identified, and an additional nine records were found through a manual search. After removing duplicate records, a total of 202 studies remained. Through evaluation of abstracts and titles, 176 studies were excluded. Subsequently, after reviewing the full text, a final selection of five studies was included in this review.

Clinical Studies of a Low FODMAP Diet and Patients Undergoing Cancer Treatments

The clinical studies examining the effects of a low FODMAP diet on cancer patients undergoing treatment are summarized in Table 1. Schaefer et al. investigated the effect of a low FODMAP diet on rectal gas and volume in prostate cancer patients undergoing radiotherapy. Twenty-five patients were randomly assigned to an "intervention group” in which they were instructed to follow a low FODMAP diet throughout their radiation treatment, while 25 patients were assigned to a "control group" in which they were not. The findings demonstrated that rectal gas and volume were considerably reduced during radiation for individuals following the low FODMAP diet compared to the control group. The study concluded that a low FODMAP diet might be beneficial in reducing rectal toxicity during radiotherapy in prostate cancer patients. High patient adherence rates to the reduced FODMAP diet dramatically reduced rectal gas and volume [26].

Another study by Holma et al. examined the correlation between consuming FODMAP and GI symptoms in patients receiving adjuvant treatment for colorectal cancer. The study included 52 patients who maintained a four-day food diary and recorded any symptoms experienced during the treatment. The quantity of FODMAP-rich food consumed was categorized as high or low. The findings indicated that patients with high consumption of FODMAP-rich foods had a higher likelihood of experiencing diarrhea than those with low consumption. Additionally, individuals who consumed high quantities of lactose-rich and other FODMAP-rich foods were more than four times as likely to develop diarrhea compared to those with low consumption of these foods. The study concluded that there was a significant association between total FODMAP intake and diarrhea symptoms, suggesting that reducing FODMAP intake could potentially alleviate diarrhea during chemotherapy [27].

In a small pilot study conducted by Larsen et al. [28] in 2018, the potential benefits of a low FODMAP diet on symptoms and health-related quality of life in patients with radiation-induced enteropathy (RE) were investigated. These patients had a history of pelvic cancer treated with radiation and presented with IBS-like symptoms.

The study included 11 female patients with RE-related IBS symptoms. Following the low FODMAP diet for four weeks, a significant reduction in IBS symptoms, as evaluated by the IBS Symptom Severity Scale (IBS-SSS) and IBS symptom questionnaire (IBS-SQ), was observed. Additionally, health-related quality of life improved based on Nepean dyspepsia index (SF-NDI) and short form 12 health survey (SF-12) scores. Despite reporting the diet as burdensome, the study successfully achieved a reduction in FODMAP intake. The study concluded that the low FODMAP diet may alleviate symptoms and improve health-related quality of life in patients with RE. However, the authors suggested the need for further controlled studies with larger sample sizes to verify these results and potentially incorporate the low FODMAP diet into the management strategy for RE.

The study acknowledged limitations, including recruitment bias, the absence of a control group, and the potential for biases in the questionnaires used [28].

Soto-Lugo et al. [29] conducted a randomized clinical trial that included 26 patients with cervical or endometrial cancer. The researchers evaluated the effectiveness of a low FODMAP diet in reducing acute GI toxicity during pelvic external beam radiotherapy (EBRT) in women with gynecological tumors. Specifically, the study aimed to detect a decrease in grade 1-2 acute GI toxicity in the standard diet group to 25% of grade 1-2 acute GI toxicity in the low FODMAP diet group. The low FODMAP diet group showed lower end-of-treatment symptom scores and less performance status deterioration. Adherence to the diet was reported by 85% of patients. The study concluded that implementing a low FODMAP diet during pelvic EBRT is a cost-effective measure that reduces symptom deterioration in cervical cancer patients.

The findings suggested that the low FODMAP diet could potentially be used alone or in combination with other measures to decrease GI toxicity and could be implemented in various healthcare settings. However, the study acknowledged the need for long-term follow-up to assess the diet's impact on chronic toxicity and the necessity for further research with a larger number of patients to establish its role in reducing severe toxicity during EBRT [29].

Finally, a recent prospective cohort study in 2023 was conducted to evaluate the efficacy of dietary intervention in patients with chronic GI sequelae resulting from treatment of cancer in the pelvic organs, where medical treatment alone was insufficient to alleviate symptoms. A total of 88 patients participated in the study. The main dietary interventions included a low-fat diet (50% of patients), modification of dietary fiber content (33%), a low FODMAP diet (20%), a gluten-free diet (1%), and other dietary advice (7%). To assess the impact of dietary intervention, the researchers used validated questionnaires to measure GI symptoms and quality of life before and after the intervention. The results showed significant improvements in quality of life, bowel function, stool frequency, constipation, incomplete rectal emptying at defecation, and performing usual activities compared to baseline [30].

Discussion

The low FODMAP diet has been primarily studied in the context of GI disorders. It has been proposed that a low FODMAP diet might improve symptoms related to IBS and IBD [20,23,24]. The currently available research has yielded useful insights on possible associations between the low FODMAP diet and cancer outcomes. For example, the observational studies (Table 1) by Schaefer et al. [26] and Holma et al. [27] indicate that a low FODMAP diet may potentially ameliorate symptoms and reduce side effects in some cancer patients undergoing treatment. Specifically, a low FODMAP diet has been found to be beneficial for reducing the incidence of rectal toxicity during radiotherapy for patients with prostate cancer and reducing diarrhea associated with chemotherapy [29,30]. Soto-Lugo et al. [29] found that a low FODMAP diet reduced symptom deterioration and improved performance status in women with gynecological tumors undergoing pelvic EBRT. Larsen et al. [28] found that a low FODMAP diet reduced irritable bowel syndrome symptoms and improved health-related quality of life in patients with RE. Whelan [31] postulated that FODMAP facilitate the delivery of fermentable substrate and water to the proximal colon, thereby providing a theoretical rationale for the relief of symptoms observed when individuals with GI disorders restrict their FODMAP intake [31].

**Table 1:** Observational studies of the low FODMAP diet in patients with cancer undergoing treatment.

Study	Study design	Participants recruited for the study	Inclusion and exclusion criteria	Dietary intervention	Outcome measures	Key results
Schaefer et al. (2020) [26]	Prospective pilot study	50 patients with prostate cancer undergoing radiotherapy.	Inclusion: Histologically confirmed prostate adenocarcinoma, age ≥ 18 years, can provide informed consent. Exclusion: Contraindications to MRI, previous pelvic surgery, inflammatory bowel disease, lactose intolerance, and use of antibiotics or probiotics within 4 weeks prior to study entry.	25 patients were assigned to the intervention group (IG) and followed a low FODMAP diet. 25 patients were assigned to the control group (CG).	The classification of rectal gas is based on a semiquantitative score (ranging from 1 to 5) and the measurement of rectal volume.	The frequency distribution of gas scores dramatically differed between the two groups, with the IG having significantly lower scores. Rectal volume was smaller in the IG (64.28 cm^3^, 95% CI 60.92–67.65 cm^3^, SD 28.64 cm^3^) compared to the CG (71.40 cm^3^, 95% CI 66.47–76.32 cm^3^, SD 40.64 cm^3^) (p = 0.02).
Holma et al. (2020) [27]	Observational study	52 colorectal cancer patients treated with adjuvant therapy.	Patients diagnosed with stage II or III colorectal cancer underwent curative surgery and were scheduled for adjuvant 5-fluorouracil chemotherapy. The study excluded patients with previous or concurrent malignancy, previous chemotherapy, other serious illnesses, or malabsorption syndromes.	The consumption of FODMAP-rich meals is measured in portions and separated into two categories.	Diarrhea frequency.	Patients with a high intake of FODMAP-rich meals were more likely to experience diarrhoea than those with a low intake (OR, 2.63; p = 0.04). Patients with a high intake of lactose-rich and other FODMAP-rich meals had a greater than fourfold increased risk of having diarrhoea compared to those with a low intake of both (OR, 4.18; p = 0.02).
Soto-Lugo et al. (2017) [29]	Single-center, prospective, randomized clinical trial	26 Patients diagnosed with cervical or endometrial cancer.	Eligible patients were between 18 and 70 years old, had histopathological confirmation of diagnosis, and were candidates for radical or adjuvant treatment with EBRT with or without concomitant chemotherapy. Patients with prior pelvic radiotherapy, inflammatory bowel disease, severe comorbidities, or distant metastasis were excluded.	The low FODMAP diet was compared to a standard Mexican diet, with adherence to the diet being assessed weekly.	The study assessed symptoms, weight, performance status, and quality of life at the beginning and end of EBRT, as well as the degree of gastrointestinal toxicity weekly according to the National Cancer Institute (NCI) v4.03 scale.	The low FODMAP diet group showed a lower end-of-treatment symptom score and lower ECOG mean deterioration compared to the standard diet group. The study did not find any significant factors associated with the presence of grade 3 gastrointestinal toxicity. Excellent adherence to the low FODMAP diet was reported in 85% of patients.
Borre et al. (2023) [30]	Prospective cohort study	88 survivors of cancer in the pelvic organs who had undergone surgery and/or radio-/chemotherapy.	The study included patients who were survivors of cancer in the pelvic organs and had undergone surgical and/or radio-/chemotherapy treatment. The main symptoms observed in the patients included bile acid malabsorption (BAM) and small intestinal bacterial overgrowth (SIBO).	The main dietary interventions were a low-fat diet (n = 44; 50%). Modification of dietary fiber content (n = 19; 33%). Dietary restrictions with a low FODMAP (n = 18; 20%). Gluten-free diet (n = 1; 1%). Other dietary advice (n = 6; 7%).	Gastrointestinal symptoms, including bowel function, stool frequency, constipation, incomplete rectal emptying at defecation, and quality of life.	The study found that dietary intervention improved quality of life (EQ5D scale) (p < 0.01), bowel function for the last four weeks (p < 0.02), stool frequency (p < 0.03), constipation (p < 0.05), incomplete rectal emptying at defecation (p < 0.02), and performing usual activities (p < 0.0) compared to baseline.
Larsen et al. (2017) [28]	Open non-controlled pilot study	11 female patients with radiation-induced enteropathy (RE) after cancer treatment.	Inclusion criteria include female patients with a history of pelvic cancer treated with radiation and with IBS-like symptoms.	Low FODMAP diet for four weeks.	IBS severity symptoms and health-related quality of life: the severity and frequency of abdominal pain, bloating, bowel habits, and interference with daily life.	IBS symptoms improved significantly based on the mean total score of IBS-SSS and IBS-SQ, which changed from 310.2 ± 60.7 to 171.4 ± 107.2 (p = 0.001) and 27.4 ± 4.1 to 15.7 ± 10.1 (p = 0.002). HRQOL improved based on SF-NDI total score (30.5 ± 9.4 to 18.3 ± 8.2, p = 0.001) and based on mental (p = 0.047) and physical (p = 0.134) score of SF-12.

In addition, earlier investigations have shown possible pathways by which FODMAP might elicit symptoms in patients with functional bowel disorders. One hypothesis suggests that the fermentation of FODMAP by gut bacteria produces gas, leading to bloating, diarrhea, and abdominal discomfort [31]. Another hypothesis suggests that the intake of FODMAP may impact the composition and function of the gut microbiota, resulting in immune activation and inflammation [32]. Furthermore, FODMAP may affect GI sensorimotor function through changes in neural and hormonal signaling [32].

However, the five studies reviewed shed light on the potential benefits of a low FODMAP diet for cancer patients undergoing various treatments [26-30]. Collectively, the findings suggest that reducing FODMAP intake may have a positive impact on reducing GI symptoms and improving patients' quality of life [26-30]. However, it is important to note the limitations of these studies. The existing research has mostly relied on a small number of patients (ranging from 11 to 88 patients) [26-30], larger randomized controlled trials and well-designed trials are still needed to validate results. Diet adherence was assessed through self-report and could be unreliable. Many studies had a short duration of intervention or follow-up period, limiting the ability to assess the long-term effects, safety, and sustainability of the low FODMAP diet [27,28,30]. Differences in cancer types, treatments, and outcomes make comparisons difficult.

Selection bias may have influenced pilot and observational studies lacking control groups, such as Schaefer et al. [26], Larsen et al. [28], and Borre et al. [30], making it challenging to determine if the observed effects were solely due to the low FODMAP intervention or other factors. Finally, Borre et al. [30] study included multiple dietary interventions, and it becomes challenging to determine the specific impact of the low FODMAP diet compared to other interventions [30].

Although limited clinical research has been conducted to investigate the associations between cancer and a low FODMAP diet, some studies have explored the potential implications of this dietary approach on cancer risk. For example, a recent large prospective cohort study conducted by Debras et al. [33] investigated the relationship between FODMAP intake (total and by type) and the risk of developing cancer, including overall, breast, prostate, and colorectal cancer. The study analyzed data from the French NutriNet-Santé prospective cohort, which included 104,909 adult participants without cancer at the beginning of the study and followed them for a median of 7.7 years. Incident cancer cases were identified through validated medical records. The findings revealed that higher total FODMAP intake was associated with increased overall cancer risk (HR = 1.21; 95% CI = 1.02, 1.44; P = 0.04). There was also a significant association between oligosaccharide intake and overall cancer risk (HR = 1.10; 95% CI = 0.97, 1.25; P = 0.04], as well as an increased risk of colorectal cancer with oligosaccharide intake (HR = 1.78; 95% CI = 1.13, 2.79; P = 0.02). Among oligosaccharides, Fructus showed a higher risk of colorectal cancer (HR = 1.44; 95% CI = 0.95, 2.16; P = 0.04). However, no significant association was found between other types of FODMAP and cancer risk. Interestingly, no association was observed between dietary FODMAP intake and overall breast cancer risk in premenopausal or postmenopausal women [33].

Furthermore, FODMAP have been proposed to have a pro-inflammatory role [34], and systemic inflammation has been positively linked to various types of cancer [35]. Barbalho et al. [34] reported that a low FODMAP diet may reduce the expression of proinflammatory markers in the body such as C-reactive protein and the fecal calprotectin and may interfere with the function of the microbiome and its metabolites. A reduction in inflammation due to a low FODMAP diet might, in theory, reduce the risk of inflammation-associated cancers, but again, more research is needed to confirm this hypothesis [34]. On the other hand, the consumption of diets rich in non-digestible carbohydrates, such as FODMAP, has been found to modulate the composition of the gut microbiome, promoting overall gut health. A recent systematic review indicated that a diet low in FODMAP reduces health-associated bacteria, such as bifidobacteria, and induces shifts resembling dysbiosis [36]. It is unclear whether the benefits of the low FODMAP diet outweigh its potential limitations in the context of cancer prevention.

Safety of the Low FODMAP Diet

An evaluation of the safety of the low FODMAP diet is crucial in determining whether patients with cancer will encounter any adverse impacts. Bodini et al. concluded that the short-term use of the low FODMAP diet was quite safe for patients with inflammatory bowel diseases [25]. However, other studies have also outlined some of the safety concerns that dietitians and patients should assess. For instance, a literature review of 41 peer-reviewed studies by Catassi et al. [27] has revealed that patients who consume a lower FODMAP diet are most likely to lower their intake of calcium, fiber, iron, folate, natural antioxidants, zinc, or even vitamins B and D. It is possible that this will result in nutritional deficiencies, particularly if the diet is not carefully planned and supplemented [37]. This could be a significant concern for cancer patients due to their increased nutritional requirements. In addition, the diet may result in poor weight loss, especially in patients with cancer who may already be at risk of losing weight due to their illness or treatment.

Again, the low FODMAP diet excludes certain carbohydrates vital to the health and diversity of the gut bacteria, and it may result in a reduction of beneficial bacteria in the body. Indeed, a literature review of 72 peer-reviewed articles by Halmos et al. has pinpointed some safety concerns that might arise from a reduction of bacterial abundance and a decline in microbiota, thereby leading to an adverse impact on psychological health and nutritional well-being [38].

It is becoming increasingly apparent that the gut microbiome plays an important role in overall health, specifically in cancer, and therefore this could potentially be considered a concern.

Finally, adhering to the low FODMAP diet can be challenging and difficulted without the guidance of an expert dietitian. Schaefer et al. [26] argued that the low FODMAP diet was feasible in reducing rectal gas and rectal volume among patients with prostate cancer only if they were able to achieve excellent compliance rates. Patients who fail to adhere to the appropriate guidelines might encounter other difficulties [26]. This is a particularly challenging situation for cancer patients who are experiencing stress and fatigue because of their illness and treatment. The complexity underscores the need for adequate guidance by professionals who have a thorough understanding of the nutritional components, as well as appropriate methods of dealing with adverse events. Considering such concerns, Halmos et al. [38] recommend that patients be advised to consult with expert dietitians who are trained in low FODMAP diets to avoid serious health complications that may occur as a consequence of following a diet low in FODMAP.

## Conclusions

In conclusion, preliminary evidence suggests that a low FODMAP diet may offer GI relief for some cancer patients, given the complexity of cancer-related GI symptoms stemming from the malignancy or treatments such as chemotherapy, radiation, or surgery. Tailored dietary interventions, therefore, could become a pivotal aspect of comprehensive supportive care. However, the implementation of such dietary strategies must consider the balance between alleviating GI symptoms and meeting the nutritional needs of cancer patients, who need a nutrient-dense diet to maintain body weight and overall health. To truly establish the efficacy, safety, and optimal application of the low FODMAP diet in cancer populations, large randomized controlled trials are necessary. Further rigorous research is essential to refine the utilization of the low FODMAP diet for managing GI issues in cancer patients and to ensure it aligns with their broader dietary requirements.
